# 

**Published:** 1857-01

**Authors:** 


### CONTENTS.

ORIGINAL COMMUNICATIONS— PAGE.

Pathology of Milk Sickness; an Inaugural Thesis, presented to the Faculty of Rush Medical College, 1856. By W. H. Philips, Kenton, Ohio...... 7

Report on the Meteorological Changes and More Important Diseases Prevalent in Chicago during the Summer of 1856. By N. S. Davis, M.D. &c...... 12

SELECTIONS, ......

Results of the Quantitative and Qualitative Analysis of Homœpathic Medical Preparations. By Edward H. Parker, M.D...... 33

Editorial Correspondence of Atlanta Medical and Surgical Journal...... 39

BOOK NOTICES,......

Obstetrics—the Science and Art. By Charles D. Meigs...... 44

The Practical Anatomist. By J. M. Allen, M.D...... 44

An Introduction to Practical Chemistry; including Analysis. By John E. Bowman, F.C.S...... 45

The Physician's Prescription Book. By Jonathan Pereira, M.D. F.R.S. 45

History and Statistics of Ovariotomy, and the Circumstances under which the Operation may be regarded as Safe and Expedient. By George H. Lyman, M.D...... 46

EDITORIAL, ......

The Medical World...... 47

Effects of Alcoholic Liquors in Phthisis...... 48

Transactions of the American Medical Association for 1856...... 52

MEDICAL INTELLIGENCE,......

To the Medical Profession and Scientific Observers...... 52

